# Myocilin polymorphisms and high myopia in subjects of European origin

**Published:** 2009-01-26

**Authors:** Tetyana Zayats, Tammy Yanovitch, Rosalind C. Creer, George McMahon, Yi-Ju Li, Terri L. Young, Jeremy A. Guggenheim

**Affiliations:** 1 School of Optometry & Vision Sciences, Cardiff University, Cardiff, Wales, UK; 2 Duke University Center for Human Genetics, Durham, NC; 3 Department of Biostatistics and Bioinformatics, Duke University Medical School, Durham, NC

## Abstract

**Purpose:**

Three previous studies have tested for an association between high myopia and polymorphisms in the open angle glaucoma gene, myocilin (*MYOC*), all in subjects of Chinese ethnicity. In two of the studies, a significant association was found while in the third, there was no association. We sought to investigate the association between high myopia and polymorphisms in *MYOC* in subjects of European ethnicity.

**Methods:**

Subjects were recruited from two sites, Cardiff University in the UK and Duke University in the United States. The Cardiff University cohort was comprised of 164 families with high myopia (604 subjects) plus 112 unrelated, highly myopic cases and 114 emmetropic controls. The Duke University cohort was comprised of 87 families with high myopia (362 subjects) plus 59 unrelated, highly myopic cases. Subject DNA was genotyped with a panel of *MYOC* single nucleotide polymorphisms (SNPs) including those found previously associated with high myopia. The Cardiff cohort was also genotyped for two flanking microsatellite markers analyzed in prior studies. Association between high myopia and *MYOC* polymorphisms was assessed using the Unphased program.

**Results:**

Since there was no evidence of heterogeneity in genotype frequencies between families and singleton samples or between cohorts, both subject groups (families and unrelated subjects) from both recruitment sites were analyzed jointly for those SNPs genotyped in common. Two variants showed significant association before correction for multiple testing. These two variants were rs16864720 (p=0.043) and NGA17 (p=0.026). However, there was no significant association after Bonferroni correction. The estimated relative risk (RR) conferred by each of the *MYOC* variants was low (RR<1.5).

**Conclusions:**

Our results suggest that *MYOC* polymorphisms have a very low, or possibly negligible, influence on high myopia susceptibility in subjects of European ethnicity.

## Introduction

Myopia is a common cause of visual impairment throughout the world, and its prevalence is increasing [1-3]. The World Health Organization has listed myopia among the leading five causes of blindness [4]. Currently, there is no effective treatment to arrest myopia progression [5]. As myopia is highly heritable [6,7], the identification of genetic variants that confer susceptibility to the condition is likely to further our understanding of its pathophysiology and may make it possible to design rational therapies to thwart myopia progression.

Several highly penetrant genetic loci for non-syndromic myopia have been mapped [8]. However, none of the causative mutations has yet been found. Candidate gene association studies have led to the identification of several high myopia susceptibility genes (Table 1) including the myocilin gene (*MYOC*) on chromosome 1. Nonetheless, replication of these findings is necessary to separate true positives from false positives.

**Table 1:** High myopia susceptibility genes.

**Gene**	**Locus**	**Reference**
Myocilin (*MYOC*)	1q23	[17]
Hepatocyte growth factor (*HGF*)	7q21	[51]
Paired box gene 6 (*PAX6*)	11p13	[52]
Collagen, Type II alpha 1 (*COL2A1*)	12q13	[42]
Lumican (*LUM*)	12q21	[53]
Collagen, Type I alpha 1 (*COL1A1*)	17q21	[54]
Transforming growth induced factor (*TGIF*)	18p11	[55]
Transforming growth factor beta 1 (*TGFB1*)	19q13	[24]

*MYOC* is best known for its role in glaucoma. Mutations in *MYOC* can cause both juvenile-onset and adult-onset open-angle glaucoma [9,10]. *MYOC* consists of three exons, and it has been shown that an upstream stimulatory factor is critical for its basal promoter activity [11]. Myocilin (also known as trabecular meshwork inducible glucocorticoid response or TIGR), the protein product of *MYOC*, was discovered during studies examining proteins that could be induced upon long-term treatment of human trabecular meshwork cells (TMC) with glucocorticoids [12]. In the human eye, myocilin is highly expressed in the TMC, sclera, ciliary body, and iris with considerably lower amounts in the retina and optic nerve head. The secreted protein is present in the aqueous humor [11]. Aside from glucocorticoid stimulation, the expression of myocilin in TMC is affected by the transcription protein transforming growth factor β (TGF β), mechanical stretch, basic fibroblast growth factor (bFGF), and oxidative stress [11,13,14]. Experimental studies show that mutant myocilin isoforms found in patients with juvenile-onset glaucoma are not secreted but accumulate in the TMC where they are thought to interfere with cell functions. For example, mutant myocilin disturbs the mitochondrial membrane potential [15]. Despite intensive research efforts, however, the precise role of *MYOC* mutations in glaucoma is unclear.

In addition to glaucomatous involvement, genetic variants in *MYOC* have also been implicated in causing susceptibility to high myopia [16,17]. This involvement would be consistent with the increased frequency of myopia in patients with open-angle glaucoma [18-20], the observation (though only in a proportion of studies) that intraocular pressure (IOP) is higher in myopes than in emmetropes [21], and the identification of significant genetic linkage close to the *MYOC* locus on chromosome 1 in families with myopia from the Beaver Dam Eye Study [22]. It is also noteworthy that some factors that stimulate myocilin expression in TMC have also been implicated in the regulation of postnatal eye growth and myopia, e.g., bFGF, TGF β, and oxidative mitochondrial pathways [23-25].

Association between *MYOC* polymorphisms and high myopia was first reported in a case-control study of Chinese subjects from Singapore [16]. An initial attempt to replicate this finding using a similar case-control design in Hong Kong Chinese subjects, however, did not support the association [26]. Later, a larger, family based association study also in Chinese subjects from Hong Kong yielded a significant result [17]. In this latter study, association was found with two microsatellite polymorphisms (NGA17 at the promoter region and NGA19 at the 3′ region) and two single nucleotide polymorphisms (SNPs; rs2421853 and rs235858 at the 3′ flanking region). Herein, association between myocilin polymorphisms and high myopia was examined in two independent Caucasian subject groups.

## Methods

### Subjects

This research followed the principles of the Declaration of Helsinki. Signed, informed consents were obtained from all participants. The number of subjects participating in the study is shown in Table 2.

**Table 2:** Number of subjects in the study.

**Subject group**	**Subjects (families) participating**		**Subjects (families) analyzed**	
	**Cardiff University**	**Duke University**	**Cardiff University**	**Duke University**
Related	604 (164)	358 (86)	551 (142)	358 (86)
Cases	112	56	121	56
Controls	114	0	116	0
Total	830	414	788	414

Note that subjects for whom all relatives were excluded were reclassified as cases or controls if they met the necessary refractive criteria.

#### Cardiff University (UK) cohort

The cohort comprised of 164 families with high myopia (604 subjects) along with an additional set of unrelated individuals comprised of 112 highly myopic cases and 114 “emmetropic” controls (spherical equivalent refractive error in both eyes >−1.00 D and <+1.00 D). Subjective refraction details were obtained from the subjects’ optometrists. DNA was extracted from saline mouthwashes and mailed to our laboratory as previously described [27]. Individuals with known syndromic disorders or a systemic condition that could predispose them to myopia were excluded. All subjects were of Caucasian ethnicity (self-reported “White Europeans”). Ethical approval for the study was granted by the Cardiff University Human Sciences Research Ethics committee (Cardiff, Wales).

#### Duke University Center for Human Genetics (USA) cohort

The cohort comprised of 86 families with high myopia (358 subjects) along with an additional set of unrelated individuals comprising of 56 highly myopic cases. All subjects underwent a complete ophthalmic examination, and individuals with syndromic conditions that could predispose them to myopia were excluded. Genomic DNA was extracted from venous blood using the AutoPure LS^®^ DNA Extractor and PUREGENE™ reagents (Gentra Systems Inc., Minneapolis, MN). The study was approved by the Institutional Review Board at the Duke University Medical Center (Durham, NC).

### Molecular genetics

**Figure 1:**
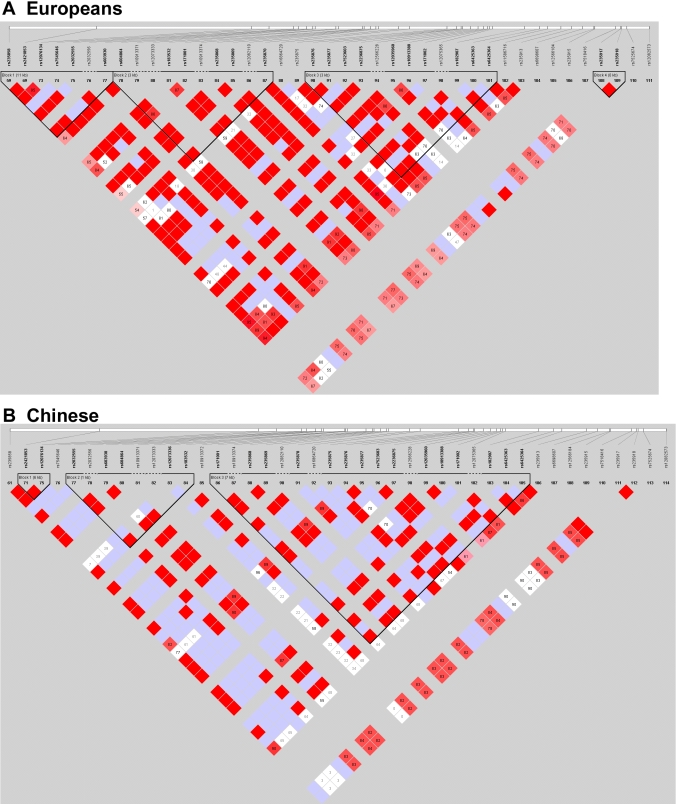
The linkage disequilibrium pattern of *MYOC* SNPs in European and Han Chinese subjects. The figure shows LD patterns in (**A**) European and (**B**) Han Chinese subjects in the HapMap database for the region running from SNP rs235858 to SNP rs12082573 on human chromosome 1 (position 142819774 to 142844986 of Genome Build 36.3 of the NCBI Human Reference sequence).

**Figure 2:**
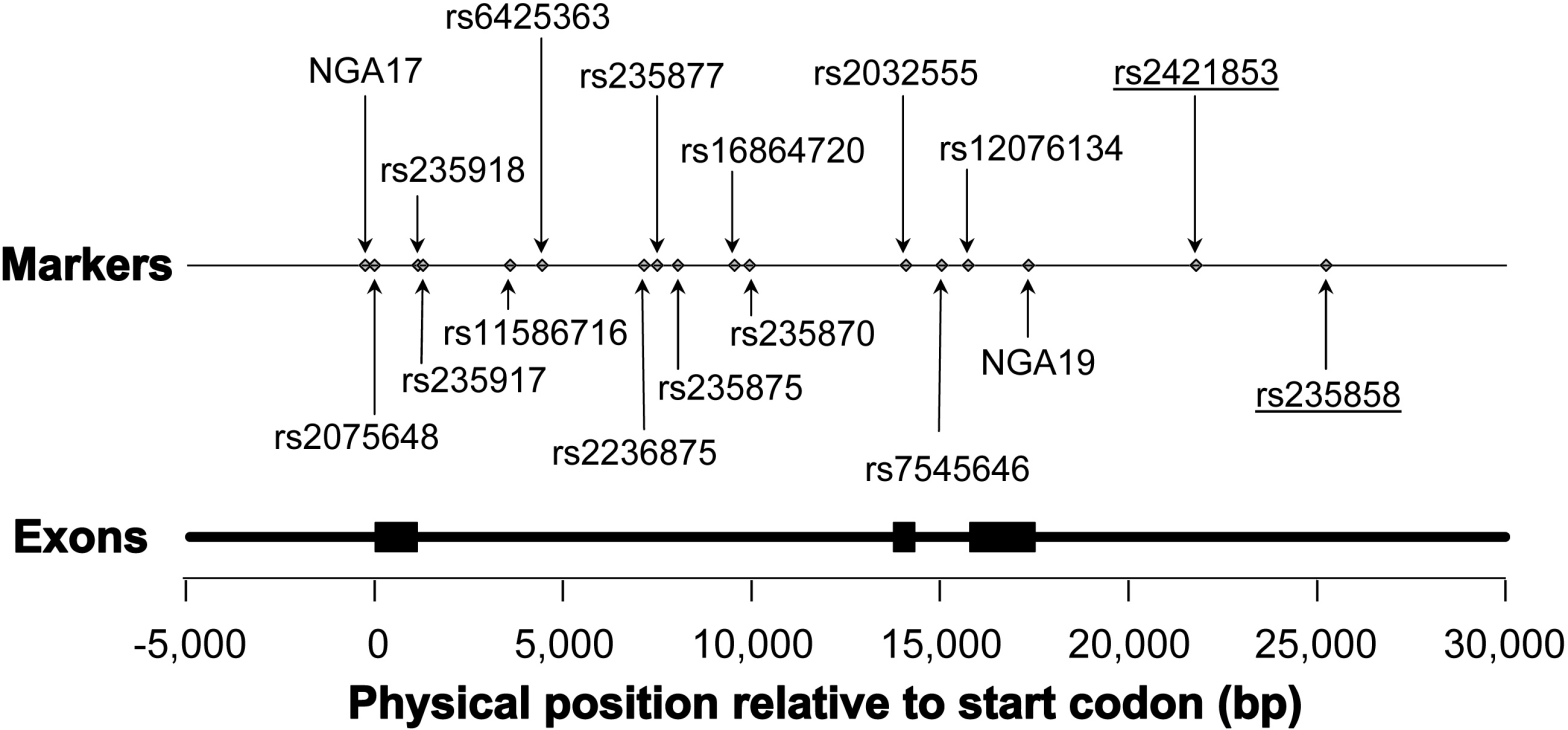
*MYOC* polymorphisms genotyped in the present study. The figure shows the positions of the polymorphisms genotyped in the present study relative to the exon structure of the MYOC gene. Exons are depicted as black rectangles, introns as intervening thick black lines. The start codon of the MYOC gene is labelled as position zero.

The HapMap database lists 25 SNPs with minor allele frequencies (MAF) greater than 5% in *MYOC* in subjects of European descent. The linkage disequilibrium (LD) structure of the gene in Europeans is shown in Figure 1A. The positions of the SNPs genotyped in this study are shown in Figure 2.

#### Cardiff University (UK) cohort

Tagging SNPs were selected using the Haploview program [28] conditional on LD (r^2^) being less than 0.8 and MAF being greater than 5% (Table 3). Genotyping was performed for 12 SNPs within and in the vicinity of MYOC, including the significant SNPs from the Tang et al. [17] study, and for two microsatellites in the untranslated regions of the gene (NGA17 at the 5' end and NGA19 at the 3' end). SNP genotyping was performed by Kbiosciences Ltd., Hoddesdon, Hertfordshire, UK. Microsatellite genotyping was performed using conventional methods [27]. Briefly, the polymerase chain reaction (PCR) mixture contained 1X HotStar PCR buffer (Qiagen Ltd., Crawley, West Sussex, UK), 1.5 mM MgCl_2_, 200 µM each dNTP, 0.3 µM of fluorescently-labeled forward primer, 0.3 µM of reverse primer, 0.1 U HotStar Taq polymerase (Qiagen Ltd), and ~20 ng genomic DNA. Amplification was achieved using PCR (35 cycles; denaturation at 94 °C for 1 min, annealing at 56 °C for 1 min, and extension at 72 °C for 1 min) after a preliminary step of 15 min at 95 °C to activate the enzyme. The primers are shown in Table 4. Amplicons were sized using an ABI Prism 310 Genetic Analyzer^®^ (Applied Biosystems, Warrington, Cheshire, UK), run on program D with Genotyper^®^ software (Applied Biosystems) used to call the alleles.

**Table 3:** Allele frequencies of *MYOC* SNPs.

**SNP name**	**SNP allele**	**Cardiff University Cohort**			**Duke University Cohort**			**Tang et al.** [17]
		**Family Founders**	**Cases**	**Controls**	**Family Founders**	**Cases**	**Controls**	**Family Founders**
rs235877	C	0.685	0.655	0.670	-	-	-	-
	T	0.315	0.345	0.330	-	-	-	-
rs235870	A	0.560	0.556	0.551	-	-	-	-
	T	0.440	0.444	0.449	-	-	-	-
rs2236875	G	0.920	0.940	0.930	-	-	-	-
	T	0.080	0.060	0.070	-	-	-	-
rs235918	A	0.353	0.366	0.347	-	-	-	-
	T	0.647	0.634	0.653	-	-	-	-
rs11586716	C	0.264	0.239	0.269	-	-	-	-
	T	0.736	0.761	0.731	-	-	-	-
rs2075648	C	0.869	0.866	0.836	-	-	-	-
	T	0.131	0.134	0.164	-	-	-	-
rs16864720	A	0.131	0.118	0.115	0.121	0.116	-	-
	G	0.869	0.882	0.885	0.879	0.884	-	-
rs7545646	C	0.087	0.074	0.078	0.100	0.116	-	-
	T	0.913	0.926	0.922	0.900	0.884	-	-
rs12076134	G	0.210	0.202	0.232	0.272	0.232	-	-
	T	0.790	0.798	0.768	0.728	0.768	-	-
rs235858	A	0.584	0.590	0.573	0.639	0.607	-	0.600
	G	0.416	0.410	0.427	0.361	0.393	-	0.400
rs2421853	A	0.232	0.217	0.245	0.300	0.277	-	0.270
	G	0.768	0.783	0.755	0.700	0.723	-	0.730
rs6425363	C	-	-	-	0.886	0.900	-	-
	T	-	-	-	0.114	0.100	-	-
rs235917	A	-	-	-	0.284	0.277	-	-
	G	-	-	-	0.716	0.723	-	-
rs235875	C	-	-	-	0.792	0.815	-	-
	T	-	-	-	0.208	0.185	-	-
rs2032555	C	-	-	-	0.239	0.277	-	-
	T	-	-	-	0.761	0.723	-	-

The values shown in the table are the SNP allele frequencies for the subjects in the Cardiff University and Duke University cohorts, who were all of Caucasian ethnicity, and for subjects in the cohort of Tang et al. [17], who were all of Chinese ethnicity.

**Table 4:** *MYOC* microsatellite primer sequences.

**Primer name**	**Primer sequence**
NGA17 forward	GCACAGTGCAGGTTCTCAA
NGA17 reverse	CCAACCATCAGGTAATTCCTT
NGA19 forward	CCGAGCTCCAGAGAGGTTTA
NGA19 reverse	CCTCAAAACCAGGCACAA

#### Duke University Center for Human Genetics (USA) cohort

Tagging SNPs were selected using SNPSelector conditional on LD (r^2^) being less than 0.8 and MAF being greater than 5% in the CEU HapMap population. Genotyping was performed for nine SNPs including the significant SNPs from the Tang et al. [17] study using TaqMan^®^ (Applied Biosystems) allelic discrimination assays (Table 3).

### Statistics

High myopia was examined as a dichotomous trait. Subjects with a spherical equivalent refractive error of less than −6.00 D (averaged between eyes) were classified as affected [17]. All other subjects were classified as unaffected. The Pedstats package [29] was used to carry out an exact test for Hardy–Weinberg equilibrium (HWE) on unrelated subjects and to check for Mendelian consistency in pedigrees. Association analyses were performed on family data only and jointly on pedigree and case-control subject data to maximize the power of association testing between *MYOC* polymorphisms and high myopia [30]. Tests were performed using the Unphased program [31], which in addition to family based assays, is able to jointly examine pedigrees and case/control samples. The recruited pedigrees from both centers included families with either one or both parents missing. However, this missingness was accounted for by Unphased, which has been shown to be free from bias in such circumstances [31]. A Bonferroni correction was applied to account for multiple testing. Importantly, the association test results for SNPs genotyped in both the Cardiff University and Duke University cohorts are only reported for combined analyses. The implications of this approach with respect to potential population stratification between subjects from the UK and USA are discussed below.

## Results

### Subjects and genotyping

The combined study population included a total of 1251 subjects (Table 2). Forty-nine subjects were excluded due to genotyping failure. The genotyping failure rate of each polymorphism is shown in Table 5. This left 293 unrelated and 909 related individuals available for association analyses: 788 subjects in the UK cohort (142 families, 121 cases, and116 controls) and 414 subjects in the USA cohort (86 families and 56 cases). Subjects for whom all relatives failed to pass our genotyping quality control threshold were classified as cases or controls if they met the necessary refractive criteria.

**Table 5:** Tests of association between *MYOC* polymorphisms and high myopia.

**Polymorphism**	**Failed genotypes (%)**	**HWE p value**	Unphased **p value (corrected p value)**	Unphased **relative risk (95% CI)**
Duke University Cohort				
rs6425363	1.5	1.00	0.57	1.15 (0.71–1.86)
rs235917	4.4	0.55	0.49	1.13 (0.79–1.59)
rs235875	2.7	0.20	0.36	1.20 (0.81–1.75)
rs2032555	3.5	0.01	Not tested due to HWE status	
Cardiff University Cohort				
rs235877	12.0	0.09	0.57	1.07 (0.84–1.37)
rs235870	9.0	0.27	0.53	0.93 (0.74–1.17)
rs2236875	10.0	0.01	Not tested due to HWE status	
rs235918	8.0	0.19	0.53	1.07 (0.86–1.34)
rs11586716	8.6	0.13	0.38	0.73 (0.84–1.44)
rs2075648	9.8	0.07	0.59	0.91 (0.64–1.28)
NGA17	0.1	0.08	0.03 (0.39)	0.70 (0.55–0.92)
NGA19	0.2	0.49	0.97	1.02 (0.82–1.26)
Combined Cohorts				
rs16864720	7.9	0.85	0.04 (0.65)	1.30 (1.004–1.73)
rs7545646	12.0	0.05	0.06	1.30 (0.98–1.8)
rs12076134	9.4	0.81	0.09	1.20 (0.97–1.48)
rs235858 *	13.0	0.86	0.87	1.02 (0.84–1.22)
rs2421853 *	13.0	0.18	0.25	1.13 (0.91–1.39)

The results shown in the table are for the analysis of the full set of subjects (i.e. families, cases and controls). For each marker studied, the table gives (column 1) the genotyping error rate, (column 2) the p-value for a contingency test examining whether the marker allele frequencies are in Hardy-Weinberg equilibrium (HWE), (column 3) the uncorrected, and in brackets the Bonferroni-corrected, p-values for an Unphased analysis examining whether the marker allele frequencies are associated with high myopia affectation status, and (column 4) the relative risk of high myopia calculated by Unphased for subjects carrying the second allele relative to carrying the first (reference) allele, along with the 95% confidence interval of the relative risk estimate. The 2 SNPs marked with an asterisk were found to be significantly associated with high myopia in the study of Tang et al. [17].

Genotyping of the two microsatellite markers, NGA17 and NGA19, revealed four alleles each. For each marker, there were three common alleles and one rare allele. The observed allele frequencies of the microsatellite polymorphisms are shown in Table 6. Since the sample size was modest, the rare allele of each microsatellite marker was combined with the allele next in size to it (allele 1 with allele 2 for both markers). Genotyping for SNP marker rs235875 failed.

**Table 6:** Allele frequencies of *MYOC* microsatellites.

**Microsatellite allele**	**Cardiff University Cohort**			**Tang et al. [**17**]**
	**Family founders**	**Cases**	**Controls**	**Family founders**
NGA17 alleles				
12 repeats	0.000	0.033	0.028	-
13 repeats	0.597	0.637	0.550	0.501
14 repeats	0.184	0.156	0.170	0.184
15 repeats	0.219	0.174	0.252	0.312
16 repeats	-	-	-	0.003
NGA19 alleles				
11 repeats	-	-	-	0.0015
12 repeats	0.000	0.014	0.000	-
13 repeats	0.342	0.344	0.400	0.218
14 repeats	0.039	0.047	0.004	0.008
15 repeats	0.619	0.595	0.596	0.711
16 repeats	-	-	-	0.060
17 repeats	-	-	-	0.0015

The values shown in the table are the microsatellite marker allele frequencies for the subjects in the Cardiff University cohort, who were all of Caucasian ethnicity, and for subjects in the cohort of Tang et al. [17], who were all of Chinese ethnicity.

### Statistical analysis

Tests for HWE showed that two SNPs, rs2236875 and rs2032555, were not in equilibrium in the unrelated subjects (Table 5). Therefore, these two markers were dropped from further analyses. Thus, association tests were performed for the remaining 15 variants, which were 13 SNPs and two microsatellites.

There was no significant heterogeneity in genotype frequencies between families and singleton samples either within or between cohorts (Table 3 and Table 6). Therefore, families and unrelated subjects were analyzed jointly [31]. Likewise, subjects recruited at Duke University and Cardiff University were analyzed jointly for those SNPs genotyped in common (i.e., ignoring potential population stratification issues). The association test results are shown in Table 5. Prior to correction for multiple testing, two variants showed significant association, rs16864720 (p=0.043) and NGA17 (p=0.026). However, neither association retained statistical significance after Bonferroni correction (Table 5). Evaluation of relative risk highlighted the same two polymorphisms, rs16864720 and NGA17, with 95% confidence intervals that did not include 1.0 (Table 5). The relative risk conferred by each of these variants, however, was low (RR<1.5). When the analysis was restricted to the family data alone, there was also no significant association between *MYOC* and high myopia (Table 7) in concordance with the joint analysis.

**Table 7:** Test of association between *MYOC* and myopia: family data only.

**Polymorphism**	**Failed genotypes (%)**	**HWE p value**	Unphased **p value (corrected p value)**	Unphased **relative risk (95% CI)**
Duke University Cohort				
rs6425363	6.30	1.00	0.59	1.18 (0.64–2.20)
rs235917	6.60	0.40	0.72	0.66 (0.60–1.75)
rs235875	5.30	1.00	0.30	1.30 (0.78–2.16)
Cardiff University Cohort				
rs235877	13.50	0.22	0.76	1.06 (0.74–1.50)
rs235870	10.15	0.10	0.58	0.91 (0.64–1.28)
rs235918	8.80	0.10	0.16	0.78 (0.55–1.10)
rs11586716	9.20	0.23	0.62	1.10 (0.77–1.54)
rs2075648	10.85	0.31	0.77	0.92 (0.55–1.57)
NGA17	0.20	0.33	0.28	0.77 (0.56–1.07)
NGA19	0.40	0.08	0.37	1.04 (0.79–1.38)
Combined Cohorts				
rs16864720	5.50	0.80	0.017 (0.289)	1.56 (1.07–2.27)
rs7545646	9.40	0.33	0.017 (0.289)	1.62 (1.07–2.46)
rs12076134	6.70	0.87	0.12	1.25 (0.94–1.67)
rs235858 *	11.00	0.33	0.69	1.05 (0.81–1.36)
rs2421853 *	10.80	0.88	0.36	1.15 (0.85–1.54)

The results shown in the table are for the analysis of the families only (i.e. cases and controls were excluded from the analysis). For each marker studied, the table gives (column 1) the genotyping error rate, (column 2) the p-value for a contingency test examining whether the marker allele frequencies are in Hardy-Weinberg equilibrium (HWE), (column 3) the uncorrected, and in brackets the Bonferroni-corrected, p-values for an Unphased analysis examining whether the marker allele frequencies are associated with high myopia affectation status, and (column 4) the relative risk of high myopia calculated by Unphased for subjects carrying the second allele relative to carrying the first (reference) allele, along with the 95% confidence interval of the relative risk estimate. The 2 SNPs marked with an asterisk were found to be significantly associated with high myopia in the study of Tang et al. [17].

## Discussion

A joint analysis of subjects from the UK and USA was performed for those SNPs that were genotyped in both groups of subjects. This pooling of subjects could potentially have given rise to a “false positive” or “false negative” association due to population stratification. However, population stratification can only give rise to a significant association between a disease phenotype and a marker genotype if the prevalence of the disease differs between the two subject groups and if the allele frequency of the marker of interest differs between the two subject groups. For high myopia, exact figures on the prevalences in Caucasian subjects from the UK and USA are lacking, but estimates suggest these rates are similar [32-34]. Furthermore, the *MYOC* polymorphisms studied here had statistically similar allele frequencies in the UK and USA subjects (Table 3 and Table 6).

In contrast to previously published significant association between *MYOC* and high myopia in subjects of Chinese ethnicity [16,17], this study suggests that there is no such relationship in subjects of Caucasian ethnicity. The ethnic difference of the respective study populations is an appealing explanation for these discrepant findings. Different populations may exhibit differences in allele frequencies and linkage disequilibrium patterns at specific loci (Figure 1 and Table 6). Thus, the role of *MYOC* in high myopia in Chinese subjects may be dissimilar to that in Caucasians.

An alternative explanation could be the power of association analyses. The estimated relative risk of the genetic variants examined here was less than 1.5, which suggests that the power of this study would be approximately 75% [35]. On the other hand, Tang et al. [17] investigated a smaller sample size (557 individuals in 162 nuclear families) and reported a relative risk greater than 1.5 for two significant SNPs (rs235858 and rs2421853). To gain 80% power, a family based association study of a variant with relative risk greater than 1.5 and allele frequency of 0.5 would need approximately 200 subjects under an additive model and approximately 1100 subjects under a dominant model [36].

A final potential reason for our failure to detect an association between *MYOC* polymorphisms and high myopia is that *MYOC* may not in fact be a high myopia susceptibility gene (i.e., the significant associations reported previously [16,17] could have been false positive findings). Several studies have suggested that many of the other high myopia genetic association results that have been published are likely to be false positives [37-48]. Moreover, candidate gene based association studies for other disorders have also yielded numerous false positive findings over the years [49].

The fact that *MYOC* polymorphisms are implicated in both myopia and glaucoma is intriguing, especially in light of the higher-than-chance co-occurrence of myopia and glaucoma seen in many studies [18-20]. Nonetheless, the high expression of myocilin in the TMC [50] is easier to reconcile with the role of *MYOC* polymorphisms in glaucoma than in myopia. Furthermore, the current evidence suggests that those *MYOC* gene variants that confer an increased risk of open angle glaucoma are different from those that increase susceptibility to myopia. In this respect, the association of *MYOC* variants with both conditions may be coincidental.

In conclusion, this study found no evidence to support a significant association between *MYOC* polymorphisms and high myopia in Caucasian subjects from the UK and USA.
